# Improved temporal speckle contrast model for slow and fast dynamic: effect of temporal correlation among neighboring pixels

**DOI:** 10.1117/1.JBO.30.7.076007

**Published:** 2025-07-18

**Authors:** Julio Cesar Juarez-Ramirez, Beatriz Coyotl-Ocelotl, David Ivan Loaiza-Toscuento, Teresita Spezzia-Mazzocco, Bernard Choi, Ruben Ramos-Garcia, Juan Pablo Padilla-Martinez, Julio Cesar Ramirez-San-Juan

**Affiliations:** a Instituto Nacional de Astrofísica, Óptica y Electrónica, Departamento de Óptica, Puebla, México; b University of California, Beckman Laser Institute and Medical Clinic, Department of Surgery, Irvine, California, United States; c Benemérita Universidad Autónoma de Puebla, Instituto de Ciencias, Puebla, México

**Keywords:** speckle, contrast, laser speckle imaging, laser speckle contrast imaging, laser speckle contrast analysis, correlation, blood flow, *Escherichia coli*

## Abstract

**Significance:**

Speckle contrast analysis, whether spatial or temporal, is a valuable optical technique extensively utilized in medical and engineering domains owing to its simplicity, affordability, and noninvasive nature. It relies on statistical analysis of the dynamic speckle pattern produced by the sample under examination, offering insights into the sample’s dynamics. However, challenges persist in precisely measuring temporal speckle contrast, particularly for slow dynamic samples. Existing mathematical models fail to accurately reflect the experimental data, which could result in misinterpretation of the analyzed results.

**Aim:**

To overcome these constraints, we present a mathematical model that incorporates the correlation between adjacent pixels. We specifically concentrate on temporal correlation, i.e., the relationship between neighboring frames, to compute the temporal speckle contrast.

**Approach:**

We theoretically replicate the statistical analysis typically conducted to compute temporal speckle contrast in a series of consecutive raw speckle images. Unlike previous models, our calculations account for the potential correlation between neighboring pixels across successive frames. To validate this model, we apply it to the analysis of the dynamics of *Escherichia coli* ATCC 25922 colonies.

**Results:**

By considering the probable temporal correlation between neighboring pixels, the proposed model notably improves the precision of temporal speckle contrast measurements, particularly for slow dynamic samples. Analytical expressions for the contrast are derived, incorporating both Gaussian and Lorentzian correlation functions, which exhibit excellent agreement with experimental findings conducted on *E. coli* colonies. Conversely, for fast dynamic samples where neighboring pixels lack correlation, our model aligns with the outcomes of the previously reported models.

**Conclusions:**

The proposed model is well-suited for computing temporal contrast in both slow and fast dynamics, rendering it applicable to a wide range of biological and industrial systems.

## 1 Introduction

Interaction of coherent laser light with rough surfaces and scattering media, such as biological tissues produce a random interference pattern usually known as laser speckle. This interaction with stationary and moving scatterers, such as tissue structures and red blood cells, produces speckle patterns that are either static or dynamic.

Interpreting the intensity fluctuations within these patterns across spatial, temporal, or combined domains is challenging but essential for extracting meaningful information about the sample dynamics. Dynamic speckle analysis is versatile, aiding in industrial applications like monitoring corrosion on metal surfaces^1^^,^^2^ and paint drying processes,^3^^,^^4^ as well as in biological assessments, including noninvasive blood flow measurements,^5^^–^^7^ seed viability testing,^8^^–^^10^ and the study of microorganisms’ behavior.^9^^,^^11^^,^^12^

Several techniques have been developed for dynamic speckle analysis,^13^ including generalized differences,^14^ weighted generalized differences,^14^ Fuji’s method,^15^ time history of the speckle pattern (THSP),^16^ speckle contrast imaging (both spatial and temporal),^17^ and principal component analysis.^18^ Among these, laser speckle contrast imaging (LSCI) is particularly valuable for studying rapid dynamics, such as those in blood vessels, by exploiting the relationship between speckle contrast and the motion within the sample.

LSCI distinguishes between spatial (examining the spatial variations of the speckles within a single raw speckle image^13^^,^^19^^,^^20^) and temporal (examining temporal speckle variations across consecutives images^13^^,^^19^^,^^21^) variations, aiding medical studies such as skin,^22^^,^^23^ retina,^24^^,^^25^ and brain research.^19^^,^^26^^–^^28^ Spatial algorithms require spatial averaging, which yields lower spatial resolution and higher temporal resolution with rapid single-frame feedback; however, they are more sensitive to static scatterers. By contrast, a temporal algorithm offers lower temporal resolution but higher spatial resolution, making it more effective at imaging deep vessels and penetrating static layers, albeit at the expense of requiring multiple frames. These findings suggest that the choice of algorithm depends on practical demands, such as the need for real-time feedback versus accurate deep tissue imaging.

Regarding to the temporal contrast, its theoretical models struggle with slow-moving dynamics, where speckle decorrelation time $\tau_{c}$ exceeds the camera exposure time T, often misrepresent the correlation between consecutive speckle images.^20^^,^^21^^,^^29^^–^^31^ For example, when examining a series of consecutive speckle images acquired within $\tau_{c}$, these images display high temporal correlation, suggesting that the temporal contrast should approach zero. However, according to current LSCI models, the temporal contrast is expected to be close to one. This discrepancy arises because the previously reported models assume zero correlation between consecutive frames, which is only valid for fast dynamics when frames are acquired over times greater than the correlation time. Many researchers have focused on scenarios involving fast dynamics, and the mathematical model works reasonably well in such cases. However, the likely correlation between frames is crucial in accurately calculating the temporal speckle contrast.

This study proposes an improved theoretical model for LSCI that incorporates frame correlation, enhancing the accuracy of temporal speckle contrast calculations for slow dynamics. The model aligns closely with experimental results, broadening LSCI’s applicability to both rapid and slow-moving phenomena and ensuring consistency with established analytical practices for highly dynamic samples.

## 2 Theory

### 2.1 Temporal LSCI

Fercher and Briers^20^ developed single-exposure speckle photography, which links the spatial contrast of time-integrated speckle patterns to the scatterer’s speed, enabling precise monitoring of flow fields. Their method uses temporal integration of backscattered light at a single pixel, with a speckle contrast formula based on Lorentzian dynamics

$$K=\frac{\sigma }{\left\langle I\right\rangle }={\left(\frac{\tau_{c}}{2T}\{1-e^{-\frac{2T}{\tau_{c}}}\}\right)}^{1/2},$$ (1)

where σ is the standard deviation and ⟨I⟩ is the mean intensity of a set of pixels, which can be selected in various ways, for instance, from a kernel within a single image (spatial contrast)^13^^,^^19^^,^^20^ or across multiple frames for a single pixel (temporal contrast).^13^^,^^19^^,^^21^ For the spatial contrast case, if the temporal resolution is not an issue for the desired application, it is recommended to average over 15 to 30 frames to improve signal-to-noise ratio. Temporal contrast improves spatial resolution compared with spatial contrast, as it preserves the original spatial resolution of the raw data.

The original Fercher and Briers model’s omission of pixel size considerations limits its applicability for slow dynamics where scatter correlation time exceeds the exposure time, such as seed growth and cell motility. Subsequent refinements^21^^,^^29^^–^^31^ introduced a more comprehensive equation to address these limitations

$$K=\alpha^{1/2}\beta^{1/2}(M)[\rho^{2}\frac{e^{-2x}-1+2x}{2x^{2}}+4\rho (1-\rho )\frac{e^{-x}-1+x}{x^{2}}+C_{n}{]}^{1/2},$$ (2)

where $x=T/\tau_{c}$, M the pixel-to-speckle grain size area ratio, ρ is the fraction of the total light attributed to dynamic scatterers, $C_{n}$ an experimental noise parameter,^21^ α a normalization (proportionality) term that accounts for effects that reduce speckle contrast such as the light polarization (because a randomly polarized light provides a lower speckle contrast than linearly polarized light^32^), and β represents the spatial component of the contrast.^33^ In the absence of spatial correlation, β is given by^29^^,^^33^ $\beta^{1/2}(M)=\sqrt{1/M}\mathrm{erf}(\sqrt{\pi M})-(1/(\pi M))(1-e^{-\pi M})$.

Equation (2) models temporal contrast using a Lorentzian correlation distribution, incorporating factors such as camera pixel size, contributions from both stationary and dynamic scatterers, and external noise. However, Eq. (2) omits temporal correlations between frames. Despite this limitation, Eq. (2) is considered a foundational model for advancing speckle contrast studies.

This theory and the corresponding temporal algorithm generally agree for $T>t_{c}$,^34^^,^^35^ but issues arise when frames within the correlation time are highly correlated, theoretically predicting σ to approach zero and, consequently, K must approach zero. However, the model in Eq. (2) inaccurately predicts K approaching 1 (when α, β, ρ=1), which can be corrected by incorporating temporal pixel correlations in temporally adjacent frames, which we describe below.

### 2.2 Proposal for a Temporal Correlation Model

To resolve these inconsistencies and enhance the accuracy of dynamic scatterer speed measurements, we propose incorporating temporal correlation among neighboring pixels when estimating K.

**Fig. 1:**
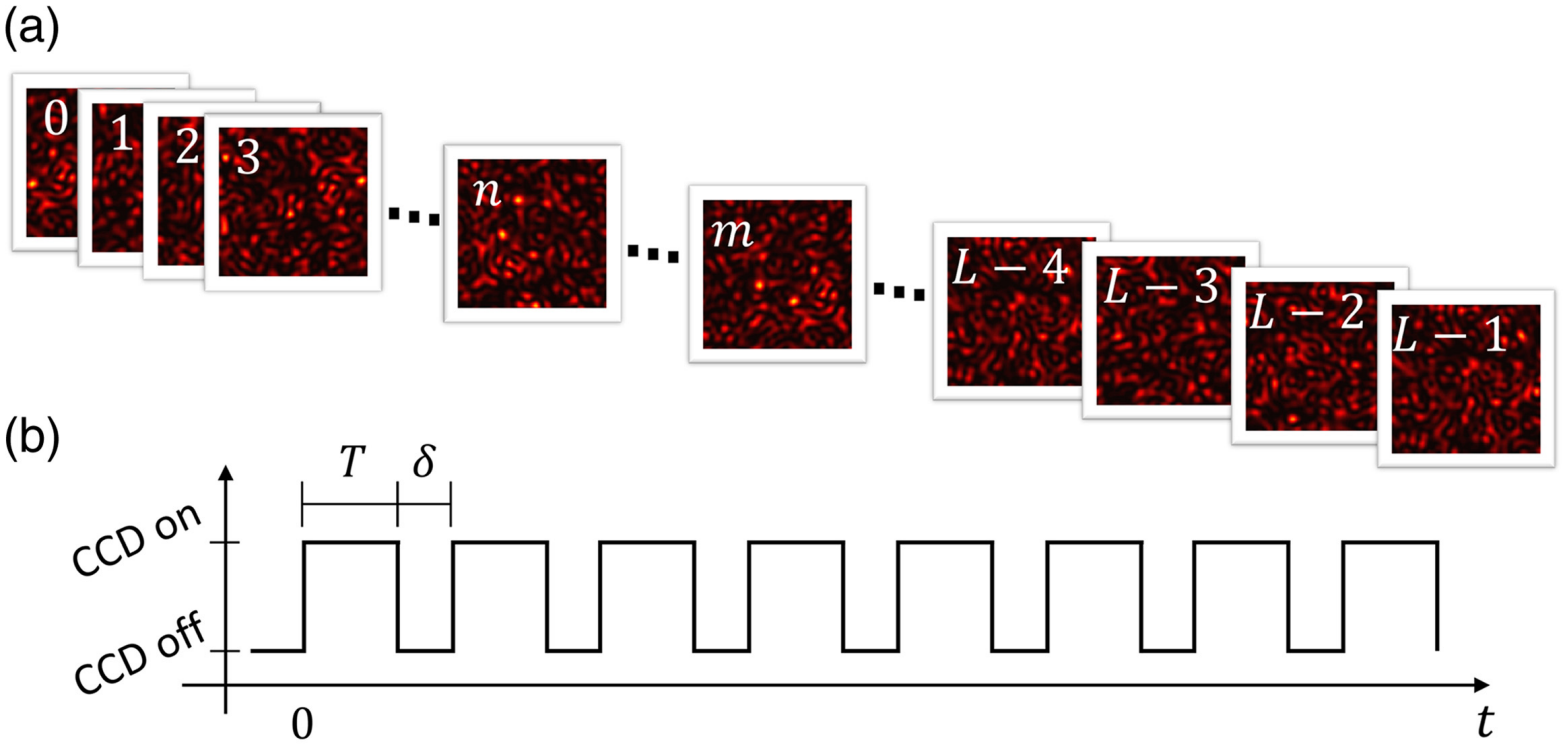
(a) Schematic representation of the set of consecutive frames. (b) Schematic representation of the camera’s acquisition time during the capture of L−1 frames.

Consider a sequence of L raw speckle images, indexed by ψ∈{0,1,2,…,L−1} [Fig. 1(a)] and captured sequentially with fixed exposure time T and δ is the time between two consecutive frames [Fig. 1(b)]. The minimum value for δ corresponds to the system’s dead time needed to process the current frame before capturing the next one. δ can typically be adjusted by the user in charge-coupled device (CCD) cameras.

S represents the number of frames within the correlation time, with S=0 indicating no inter-frame correlation; only autocorrelation within a single frame is relevant. Conversely, S=L−1 indicates correlation across all L frames. The degree of correlation varies based on the value of S.

**Fig. 2:**
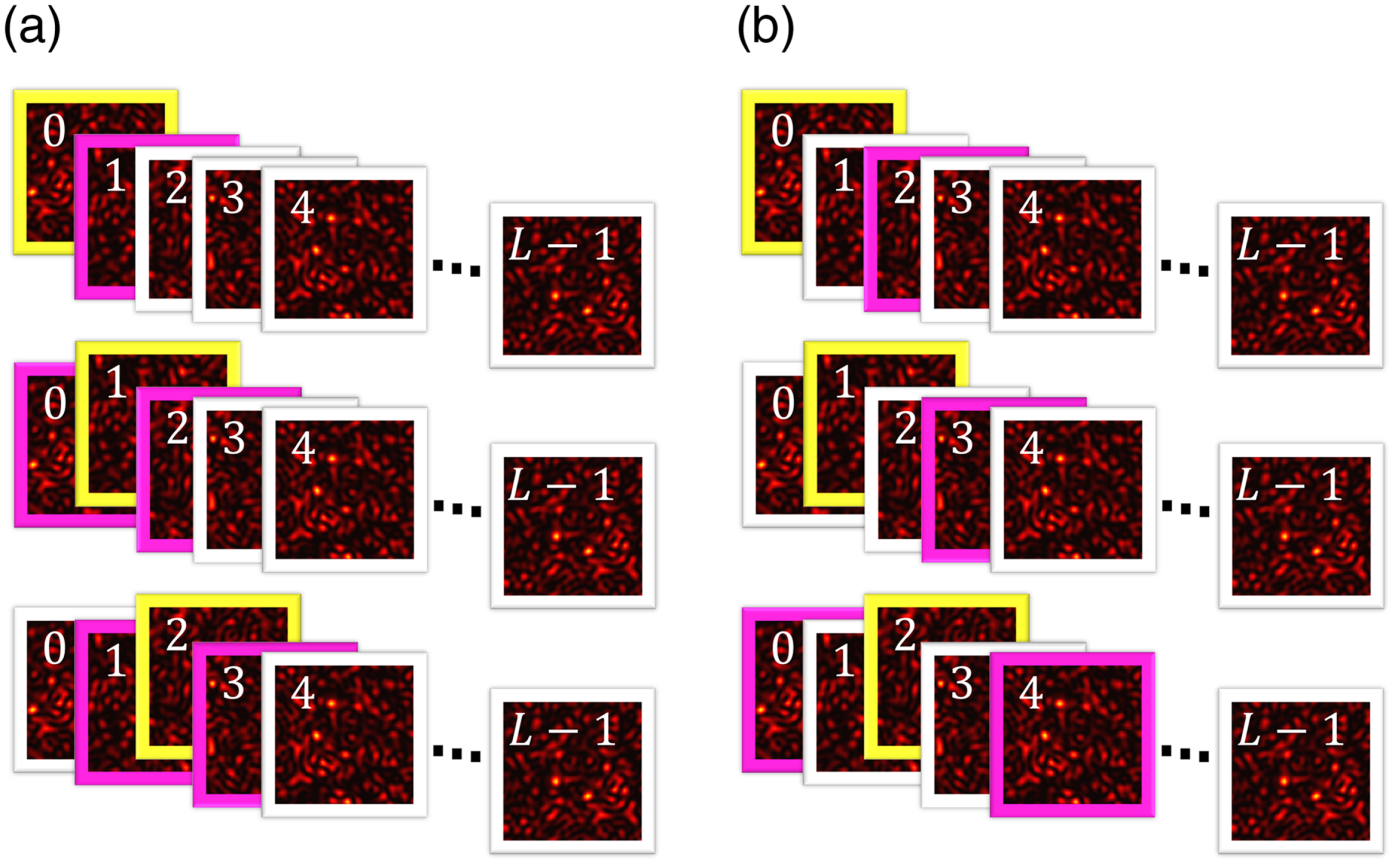
Depicts (a) a schematic illustration of the autocorrelation 1-type, between the selected frame highlighted in yellow and the highlighted in magenta. The first and last frames have only one  1-type autocorrelation, whereas all others exhibit two 1-type autocorrelations. (b) A schematic representation of the frame 2-type, again between the selected frame highlighted in yellow and the highlighted in magenta. The first two and last two frames display only one 2-type autocorrelation each, whereas all other frames show two 2-type autocorrelations.

The ψ-type autocorrelation is defined as the autocorrelation between frames separated by ψ frames, i.e., autocorrelation with a delay of (T+δ)·ψ, denoted by the correlation between the n-th and the m’th frame, such that |m−n|=ψ. The 0-type autocorrelation corresponds to the autocorrelation when the delay between frames is 0. Each frame exhibits one 0-type autocorrelation, as depicted in Fig. 2. The 1-type autocorrelation occurs between adjacent frames (with exceptions for the first and last frames), and the (L−1)-type autocorrelation denotes the correlation between the first and last frame, with only two such correlations in the sequence.

There are L autocorrelations (0-type), whereas other ψ-type autocorrelations (ψ>0) depend on the frames positions, with the number of correlations decreasing as ψ increases (see Fig. 2). The number of autocorrelations of ψ-type denoted by $\beta_{\psi }$ is

$$\beta_{\psi }=\{\begin{array}{ll} L & \psi =0,\\ 2(L-\psi ) & 0<\psi \leq L-1. \end{array}$$ (3)

Considering these likely temporal correlations between neighboring pixels in a set of speckle images acquired with exposure time T and a delay between frames of δ, the process of calculating the contrast, detailed in the Appendix, requires computing the correlation for all possible combinations of frames, given by $\frac{1}{L^{2}}\overset{L-1}{\underset{\psi =0}{\sum }}\overset{L-1}{\underset{\psi^{\prime }=0}{\sum }}\langle I_{0},I_{\psi }\rangle$. The computation involves categorizing correlations into three types: autocorrelations $\underset{\text{central}}{\sum }\langle I_{0},I_{\psi }\rangle$ when ψ=0; lateral (correlations within the temporal window ψ≤S) $\underset{\text{lateral}}{\sum }\langle I_{0},I_{\psi }\rangle$, and outsiders (correlations beyond the temporal window of correlation ψ>S) $\underset{\text{outsiders}}{\sum }\langle I_{0},I_{\psi }\rangle$, following the notation of our spatial contrast model [33]. Previous models often neglect all correlations except for autocorrelations.

Based on the mathematical formulations provided in Appendix, the temporal contrast can be expressed as follows:

$$K_{t}^{2}(x,L,S)=\frac{1}{\nu_{0}}-\frac{2}{L(L-1)}\overset{S}{\underset{\psi =1}{\sum }}(L-\psi )\frac{1}{\nu_{\psi }},$$ (4)

where the parameter $\nu_{\psi }$ accounts for the temporal correlation between frames separated by ψ number of frames and is calculated by

$$\frac{1}{\nu_{\psi }}=\frac{1}{T^{2}}\int_{0}^{T}\int_{\psi (T+\delta )}^{(\psi +1)T+\psi \delta }g_{1}^{2}(t-t^{\prime })dt^{\prime }\;dt,$$ (5)

where $g_{1}(t-t^{\prime })$ is the temporal field correlation (first-order correlation).

The summation in Eq. (4) accounts for correlations from 1-type to S-type, but for S=0, only the autocorrelation contributes, simplifying the equation to

$$K_{t}^{2}(x,L,0)=\frac{1}{\nu_{0}},$$ (6)

where $\nu_{0}$ exclusively represents autocorrelation, derived from Eq. (5) with ψ=0, whereas the summation is valid only for S>1.

### 2.3 Gaussian Correlation Function

Knowledge of the temporal field correlation (first-order correlation) is critical to calculating the correlations between frames [Eq. (5)]. Temporal field correlation can be modeled using Gaussian or Lorentzian correlation functions. Blood flow exhibits different Reynolds numbers Re, ranging from laminar (Gaussian correlation^36^^,^^37^) in small arterioles (Re≈1) to turbulent in larger arteries (Re≈4000)^38^ (Lorentzian correlation^36^). Fluid flow through a straight pipe typically transitions from laminar to turbulent at a Reynolds number of around 2300.^39^ However, turbulent transitions may occur even at low Reynolds numbers (around 300)^38^ due to stenosis or vessel collapse.

#### 2.3.1 Analytical solutions for Gaussian correlation

Equation (5) can be solved analytically under the assumption of a Gaussian correlation,^35^ which is defined to satisfied Mandel’s definition of correlation time $\tau_{c}$, which is the characteristic time scale over which the speckle intensity pattern decorrelates to $e^{-1}\;$ due to motion or changes in the scattering medium within the sample (see Appendix for more details). The first-order temporal field correlation is expressed as

$$g_{1}(\tau )=\exp[-\frac{\pi }{2}\frac{\tau^{2}}{t_{c}^{2}}].$$ (7)

**Fig. 3:**
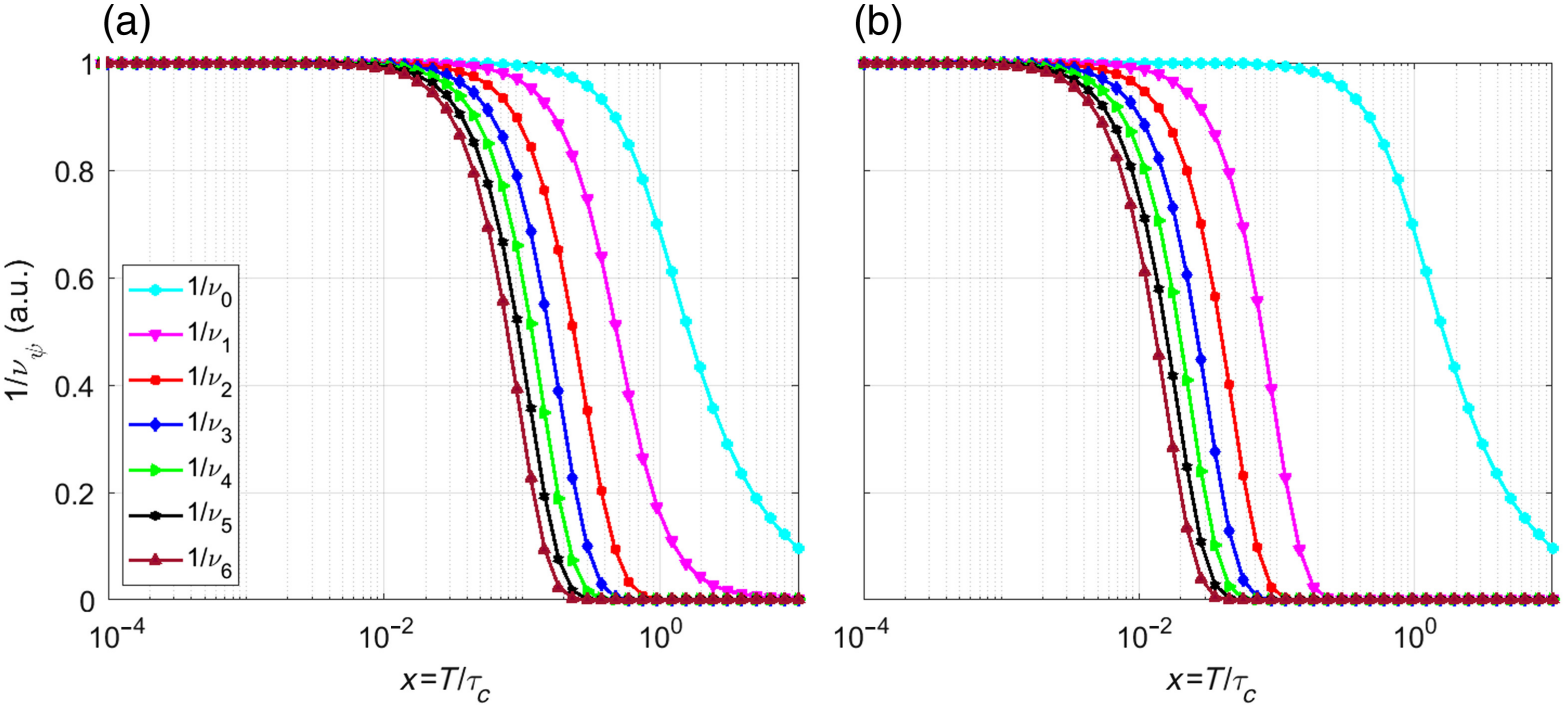
First seven Gaussian correlation factors for ψ=0 to ψ=7 considering, (a) Δ=0 and (b) Δ=5, as a function of the ratio of the exposure time and correlation time.

By substituting Eq. (7) into Eq. (5) and integrating, we obtain

$$\frac{1}{\nu_{\psi }}=\frac{1}{2x}\{(\mathrm{erf}[\sqrt{\pi }(\psi +\psi \Delta )x]-\mathrm{erf}[\sqrt{\pi }(\psi -1+\psi \Delta )x])(1-\psi -\psi \Delta )\;+(\mathrm{erf}[\sqrt{\pi }(\psi +1+\psi \Delta )x]-\mathrm{erf}[\sqrt{\pi }(\psi +\psi \Delta )x])(1+\psi +\psi \Delta )\}\;+\frac{1}{2\pi x^{2}}(\exp[-\pi (\psi -1+\psi \Delta {)}^{2}x^{2}]-2\;\exp[-\pi (\psi +\psi \Delta {)}^{2}x^{2}]\;+\exp[-\pi (\psi +1+\psi \Delta {)}^{2}x^{2}]),$$ (8)

where Δ=δ/T and $x=T/\tau_{c}$. To compare the contributions and the effect of Δ on the different correlation factors $1/\nu_{\psi }$, Fig. 3 compares the first seven correlation factors (ψ=0,1,…,7) for two scenarios: (a) no delay (Δ=0) and (b) a delay 5 times longer than the exposure time (Δ=5) emphasizing the effect of Δ>0. In systems with slow dynamics, delays often exceed the exposure time (Δ>1), whereas models that ignore correlations assume Δ=0.

When only autocorrelations are considered, as described in our previous analysis,^35^ the speckle contrast simplifies to

$$K_{t}^{2}(x,L,S)=\frac{1}{\nu_{0}}=\frac{\mathrm{erf}[\sqrt{\pi }x]}{x}+\frac{\exp[-\pi x^{2}]-1}{\pi x^{2}}.$$ (9)

Equation (9) does not incorporate delay Δ, number of frames L, or number of correlation frame S, as expected, as these factors are irrelevant in an autocorrelation-only model.

**Fig. 4:**
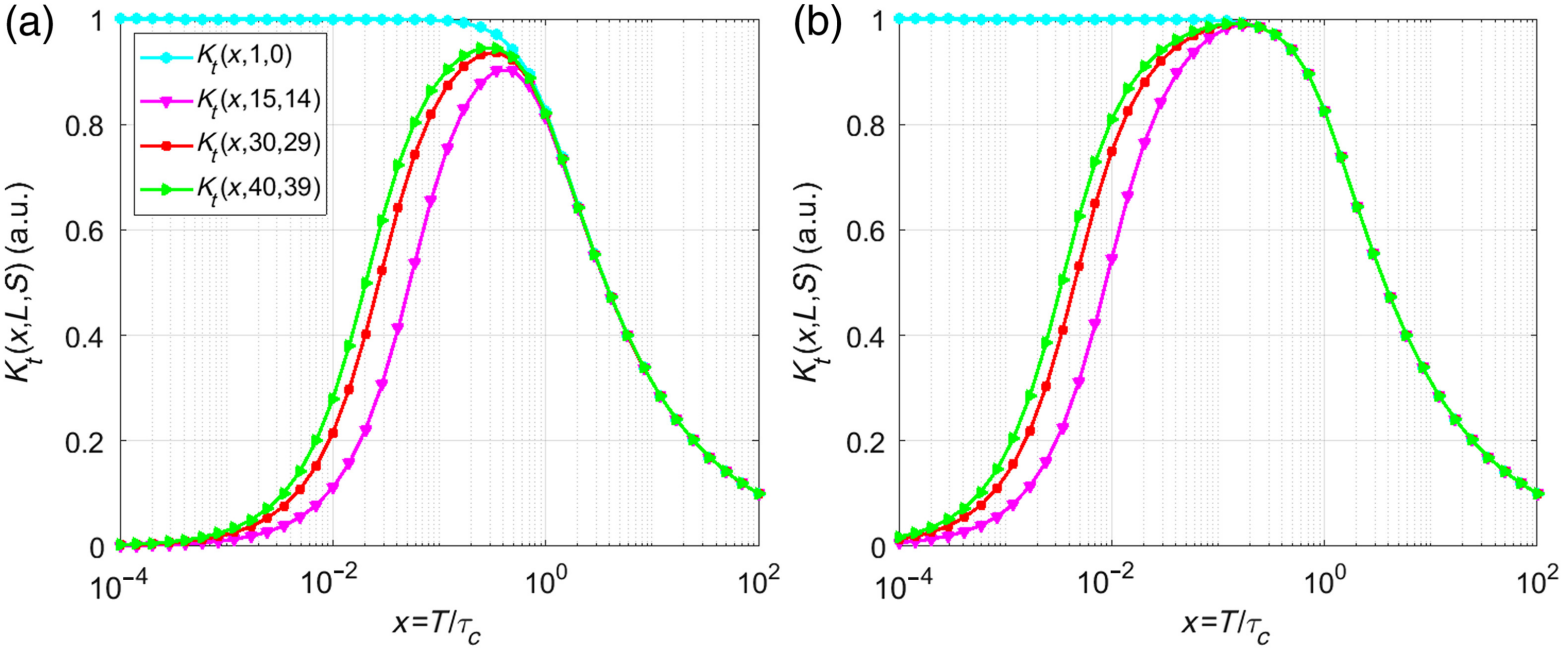
Contrast [Eqs. (4) and (8)] for Gaussian correlation and L=1, 15, 30, 40, (a) considering Δ=0 and (b) Δ=5.

Figure 4 shows temporal contrast as a function of x for L=1, 15, 30, and 40 and S=L−1, comparing models (a) without Δ=0 and (b) with (Δ=5) delays, based on Eqs. (4) and (8), respectfully. The cyan line represents a model without correlation [Eq. (2) when α, β, ρ=1), predicting a monotonically decreasing contrast from 1 to 0, ignoring stationary scatterers.

Including correlations between neighboring frames introduces unique local maxima in the contrast curves, a feature absent in models described by Eqs. (1) and (2). For x≪1 (highly correlated frames), the proposed model predicts a temporal contrast close to zero, whereas Eqs. (1) and (2) incorrectly estimate a contrast near 1. Both models agree for x>0.5.

### 2.4 Lorentzian Correlation

The Lorentzian correlation function^36^^,^^37^ is frequently used in LSCI and supports the models described by Eqs. (1) and (2).

#### 2.4.1 Analytical solutions for Lorentzian distribution

Equation (5) can also be solved analytically using a Lorentzian correlation function^35^ (see Appendix for more details)

$$g_{1}(\tau )=\exp[-\frac{\left|\tau \right|}{t_{c}}].$$ (10)

**Fig. 5:**
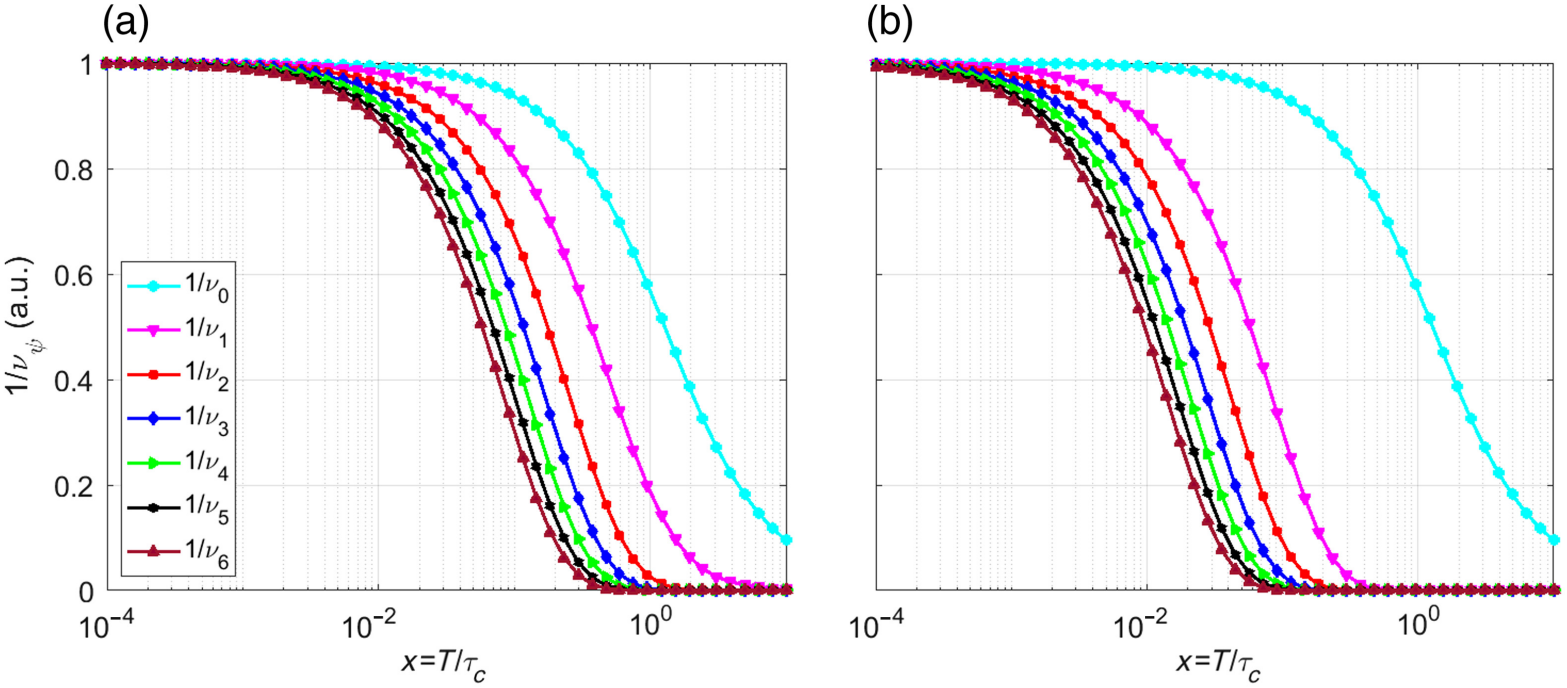
First seven Lorentzian correlation factors for ψ=0 to ψ=7 considering, (a) Δ=0 and (b) Δ=5.

Due to the absolute value in Eq. (10), Eq. (5) requires separate integrations for ψ=0 and ψ>0, leading to distinct results. For ψ=0

$$\frac{1}{\nu_{0}}=\frac{1}{2x^{2}}(2x+\exp[-2x]-1),$$ (11)

for ψ>0

$$\frac{1}{\nu_{\psi }}=\frac{1}{4x^{2}}(-2\;\exp[-2(\psi +\psi \Delta )x]+\exp[-2((\psi -1)+\psi \Delta )x]+\exp[-2((\psi +1)+\psi \Delta )x]),$$ (12)

as before, Δ=δ/T and $x=T/\tau_{c}$. Figure 5 illustrates the first seven correlation factors (ψ=0,1,…,7) for a) Δ=0 and b) Δ=5.

For the simplest case ψ=0, the analytical solution for $1/\nu_{\psi }$ recovers a well-known expression for temporal contrast,^30^^,^^34^^–^^36^^,^^40^ given as

$$K_{t}^{2}(x,L,S)=\frac{1}{\nu_{0}}=\frac{1}{2x^{2}}(2x+\exp[-2x]-1).$$ (13)

Similar to the Gaussian case, Eq. (13) excludes dependence on Δ, as expected, as it considers only autocorrelation, making inter-frame delay irrelevant.

MATLAB and Mathematica implementations for computing temporal contrast $K_{t}$ [Eq. (4)] and $1/\nu_{\psi }$ for Gaussian and Lorentzian correlations, applicable to any ψ, have been developed and are publicly accessible. The implementations can be found in the Code and Data Availability section.

**Fig. 6:**
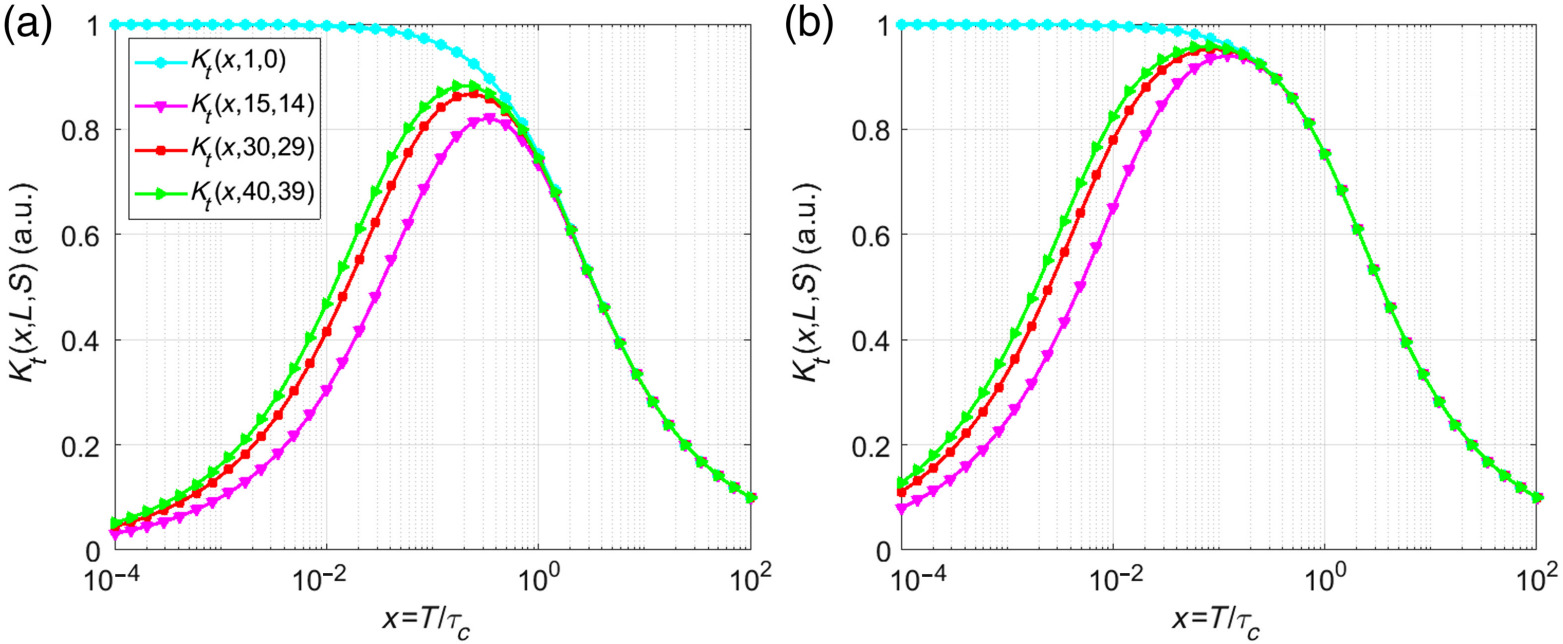
Contrast [Eqs. (4) and (8)] for Lorentzian correlation and L=1, 15, 30, 40, (a) considering Δ=0 and (b) Δ=5.

Figure 6 shows contrast values for L=1, 15, 30, 40, and S=L−1, calculated using Eqs. (11) and (12) for  Δ=0 and Δ=5, respectively. The cyan line represents a model without correlation [Eq. (11)] that excludes correlations between neighboring frames.

As in the previous case, including correlations between neighboring frames introduces distinct local maxima in contrast curves, which shift leftward compared with the Δ=0 case, a phenomenon absent in the previously reported models. For x≪1, the proposed model predicts near-zero contrast, aligning with high inter-frame correlation, whereas Eqs. (1) and (2) erroneously estimate a contrast near 1. For x>1, both models agree.

## 3 Experimental Verification

### 3.1 Experimental Setup

To validate the proposed theoretical model, we need the speckle frames to have a certain degree of correlation (slow dynamics), which depends not only on the dynamics of the object under study but also on the camera speed. In this work, we use a colony of *Escherichia coli* in a Petri dish; however, it could be used a wide variety of samples, for example, monitoring corrosion on metal surfaces,^1^^,^^2^ paint drying processes,^3^^,^^4^ seed viability testing,^8^^–^^10^ the study of microorganisms’ behavior,^9^^,^^11^^,^^12^ and detection of bacteria in liquid media^41^^,^^42^ to mention some few of them.

**Fig. 7:**
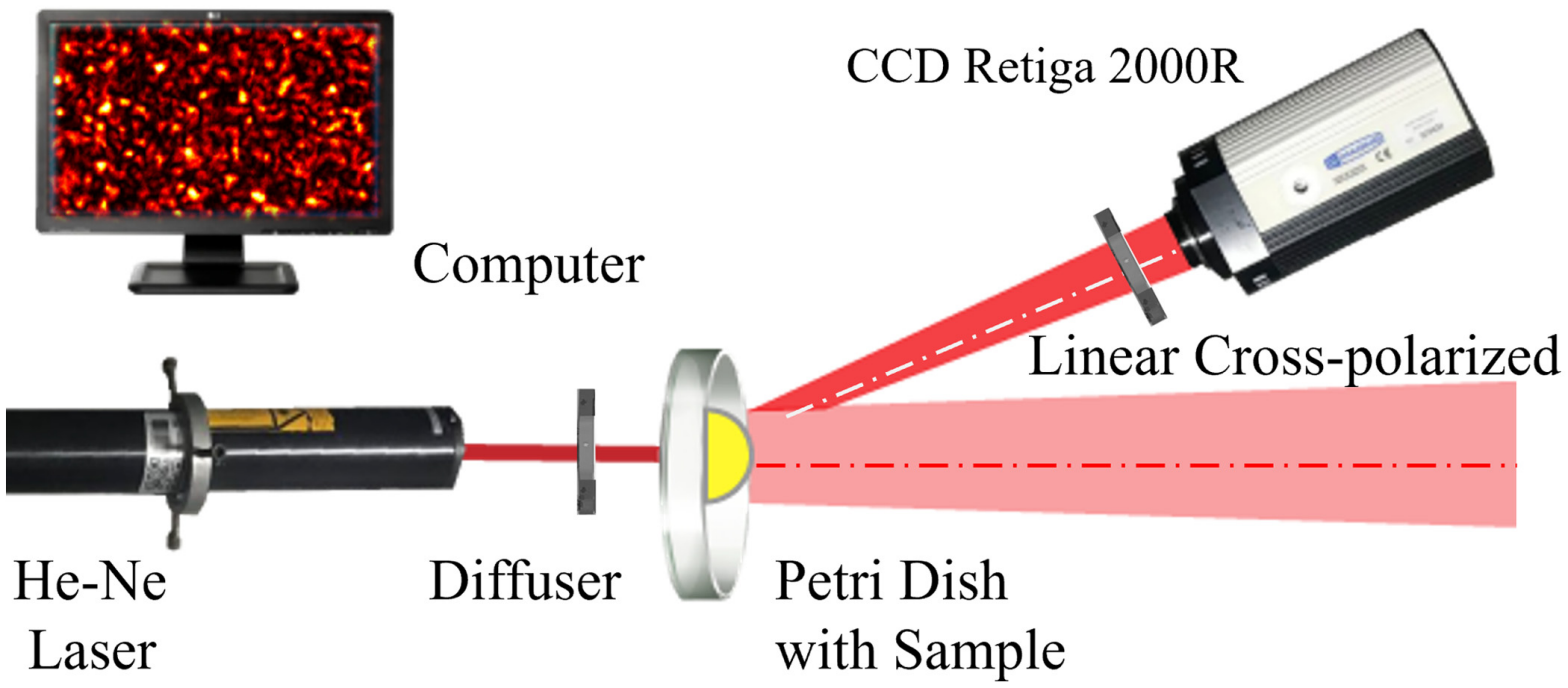
In the setup schematic, a He–Ne laser illuminated an *E. coli* sample in a transillumination geometry. The camera was positioned off-axis to prevent saturation caused by ballistic photons, which has minimal interaction with the sample and retains little information about its dynamics.

The experimental setup (Fig. 7) involved a linearly polarized He-Ne laser (Melles Griot, 20 mW, 632.8 nm wavelength), a holographic diffuser (Edmund Optics, 5-deg diffusing angle) to produce uniform illumination, a CCD camera (Retiga 2000R, 7.4  μm×7.4  μm pixel area) to capture a sequence of images and a crossed polarizer to eliminate specular reflections. The CCD camera was positioned 20 deg off the laser beam axis to avoid direct light exposure. A lens was not attached to the CCD camera.

#### 3.1.1 Biological sample

The experiment utilized *E. coli* ATCC 25922 colony, cultivated on soy dextrose agar (BD Bioxon) for 24 h at 37°C. The resulting growth was collected in a 1× phosphate-buffered saline solution (PBS) (Omnichem). Dilutions were prepared to achieve a final concentration of $5\times 10^{3}\text{ cells}/\mathrm{mL}$, and 50  μL was inoculated on a region of a $60\times 15\;{\mathrm{mm}}^{2}$ Petri dish using the spread plate method, followed by incubation at 37°C for 24 h to grow isolated colonies. Under these conditions, the *E. coli* sample exhibited dynamics significantly slower than those of blood flow.

After *E. coli* colonies were grown, the Petri dishes were positioned as shown in Fig. 7. The whole setup is on a floating table to minimize vibrations. Images were captured at room temperature (20 to 25°C) from an isolated colony obtained through serial dilution. A sequence of 901 frames was captured with an exposure time of T=1  s, resulting in x=0.002 and Δ=δ/T=0.347.

### 3.2 Experimental Results

**Fig. 8:**
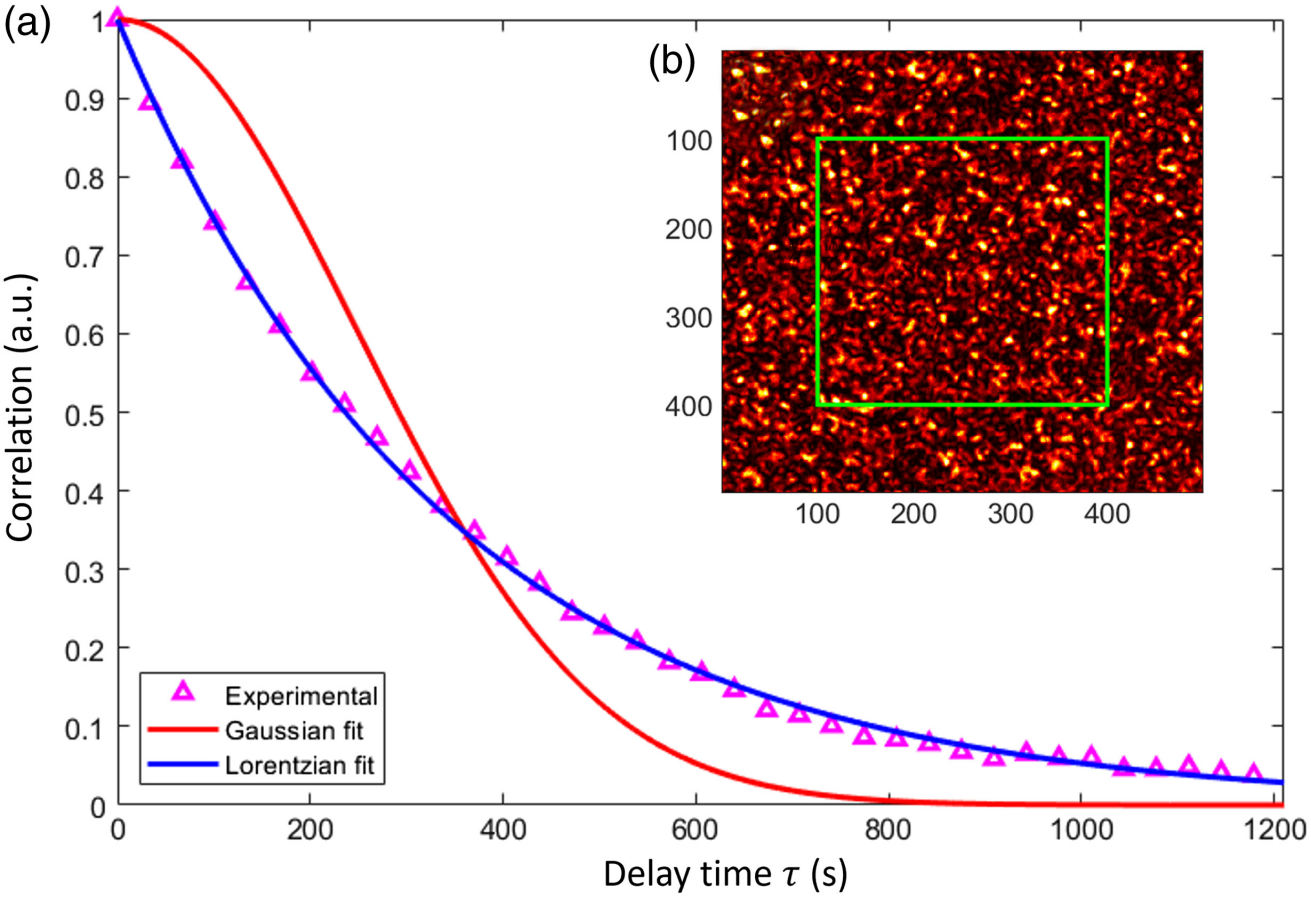
(a) Correlation between the first frame versus the rest (magenta triangles), Lorentzian (blue line), and Gaussian fit (red line). The Lorentzian correlation time for this set of frames is $\tau_{\mathrm{cl}}=340.8\;s$, whereas for the Gaussian correlation time $\tau_{\mathrm{cg}}=439\;s$. (b) Typical speckle frame and the interest region (green square) of 300×300  pixels. The exposure time is T=1  s.

The correlation time $\tau_{c}$ was calculated by fitting the correlation of the first frame against subsequent frames (magenta triangles in Fig. 8). Both Lorentzian and Gaussian correlation functions were used for fitting, represented by the red and blue lines, respectively.

The fitted Gaussian and Lorentzian correlation time was $\tau_{\mathrm{cg}}=439\;s$ and $\tau_{\mathrm{cl}}=340.8\;s$, respectively. Although calculations were made for both Gaussian and Lorentzian models, only Lorentzian results are presented, due to, as shown in Fig. 8, a superior fit to the experimental data is obtained. The Lorentzian fit indicates that the dynamic behavior of the *E. coli* colony resembles Brownian motion.^36^^,^^37^

**Fig. 9:**
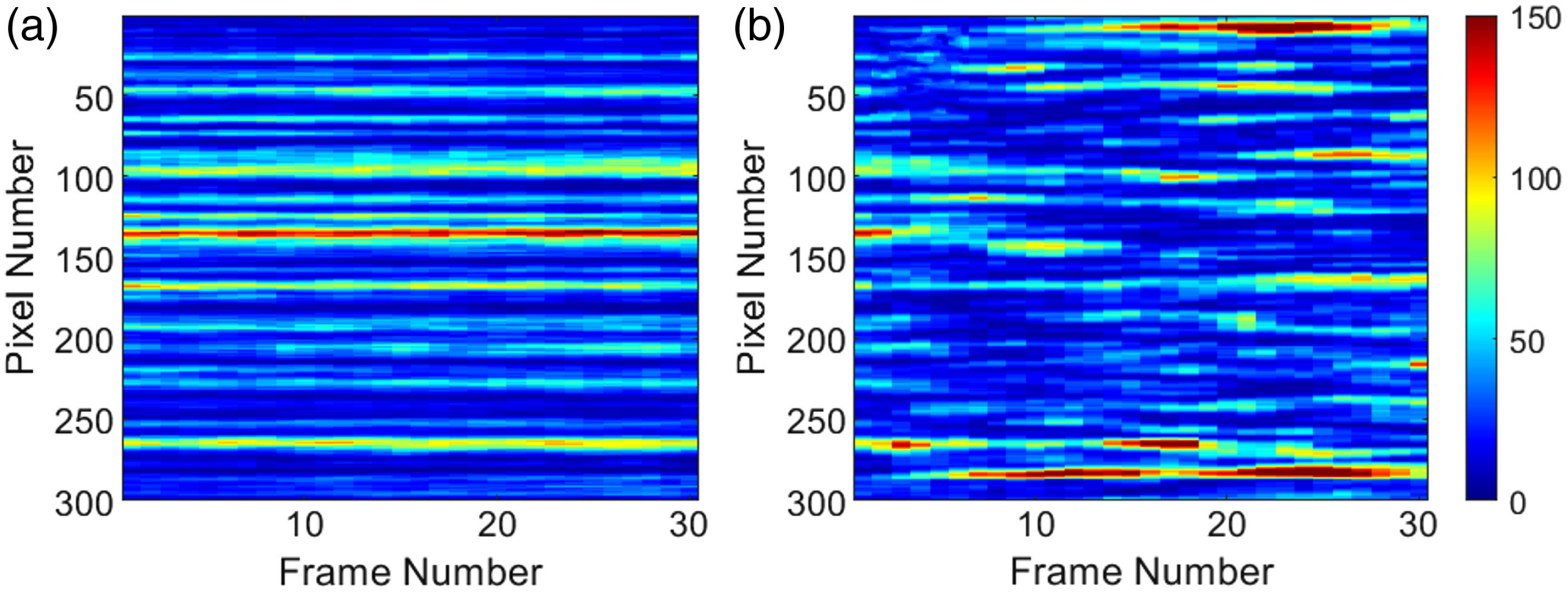
Time histories speckle pattern of the central columns was calculated for the extreme values of the experimental data: (a) x=0.0029 and (b) x=0.0880, respectively. The length of the horizontal stripes corresponds to the number of frames in which a speckle grain appears. Consequently, shorter stripes indicate faster dynamics, whereas longer stripes indicate slower dynamics.

**Fig. 10:**
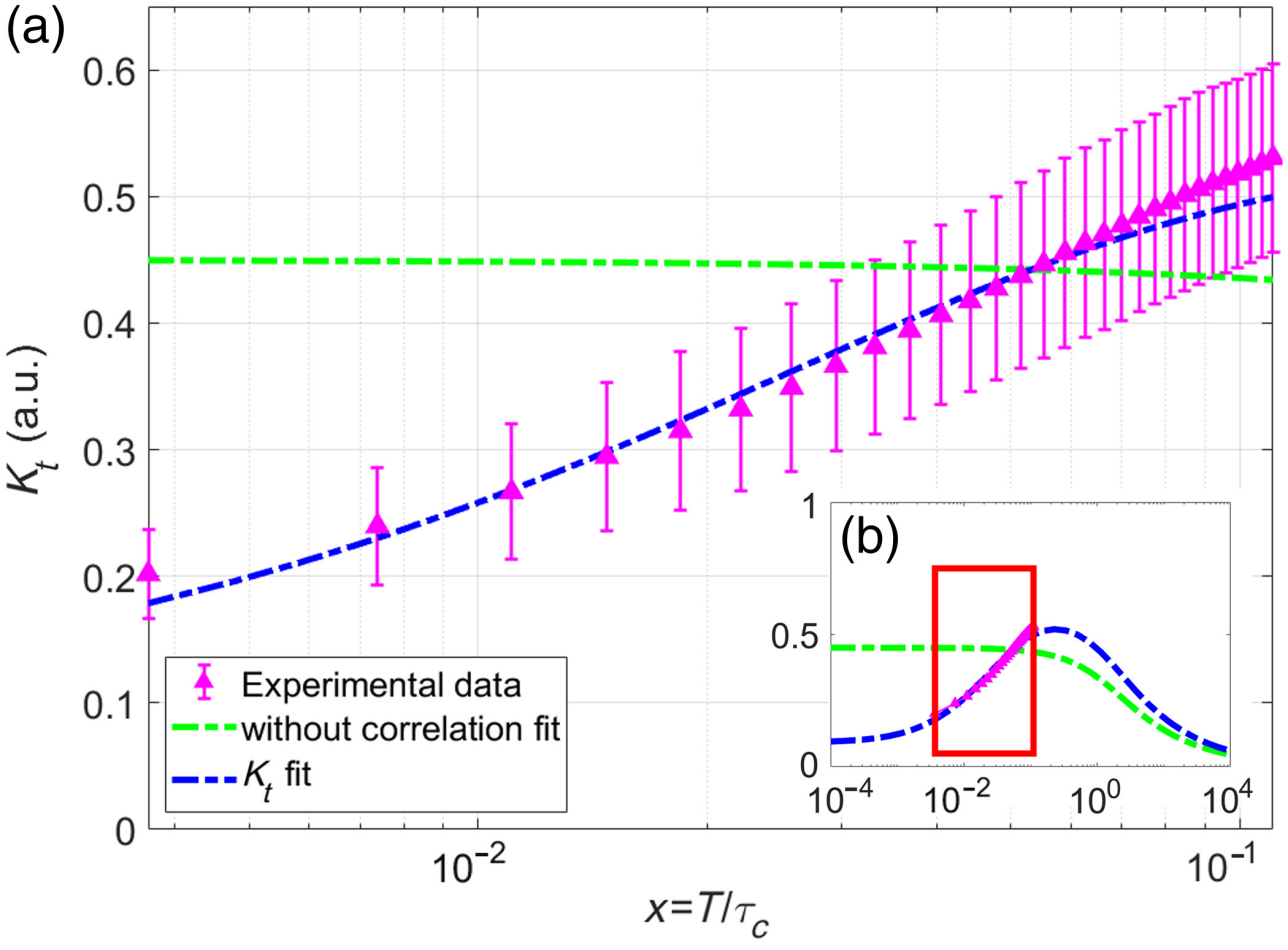
Experimental data were obtained by averaging ω consecutive frames (magenta triangles) with an exposure time of T=1  s, where ω=1,2,3,…,30. Previously reported model (green-dashed line) and our proposal (blue dashed line) were both fitted to the experimental data. The error bars correspond to the standard deviation of the contrast. (a) The experimental data are presented in a logarithmic plot, whereas panel (b) provides a zoomed-out view.

The dynamics of the living *E. coli* colony can vary due to environmental factors such as temperature, laser intensity, colony age, and nutrient availability in the Petri dish. To compare dynamics consistently, experimental data were captured with T=1  s, and L consecutive frames (1 to 30) were integrated to simulate exposure times greater than 1 s. Figure 9 illustrates the differences in dynamics for two values: (a) x=0.0029 and (b) x=0.0880 (almost extreme values from Fig. 10), through the time history speckle pattern^43^ of the central column of the experimental raw speckle images. The length of the horizontal stripes corresponds to the number of frames in which a speckle grain remains unchanged. Thus, shorter stripes indicate faster dynamics, whereas longer stripes are associated with slower dynamics; thus, Fig. 9(b) indicates a faster dynamics as those shown in Fig. 9(a).

Figure 10 shows temporal contrast for L=30 frames, with (a) presenting experimental data on a logarithmic scale and (b) offering a zoomed-out view with a logarithmic x-axis. A red rectangle highlights the region of interest. The error bars correspond to the standard deviation of the contrast.

The proposed model (blue dashed line) shows excellent agreement with experimental data (magenta triangle) for x<1, as anticipated. Moreover, the noncorrelation model (green-dashed line) deviates from the experimental data in the region $x=10^{-3}$ to $x=10^{-1}$, where the experimental data increases while the no-correlation model remains almost constant. In Fig. 10(b), it shows that both models agree for $x>10^{0}$.

## 4 Spatial-temporal Analysis

In practical applications, both spatial and temporal correlations are present; therefore, it is necessary to account for the spatial correlation among neighboring pixels within the same frame, as well as the temporal correlation across frames.

Although spatial-temporal correlation has not been considered so far, the electric field $E(\vec{r},t)$ depends on both space and time combining a static field $E_{s}$ and a fluctuating field $E_{f}$ and fluctuating fields, i.e., $E(\vec{r},t)=E_{f}(\vec{r},t)e^{-i\omega t}+E_{s}(\vec{r})e^{-i\omega t}$, the intensity autocorrelation of the fluctuating field $g_{1,f}(\Delta \vec{r},\tau )$ can be expressed as a separable product in space and time

$$|g_{1,f}(\Delta \vec{r},\tau )|=|g_{1,s}(\Delta \vec{r})||g_{1,f}(\tau )|,$$ (14)

where $g_{1,f}$, $g_{1,s}$ are the intensity autocorrelation of the fluctuating and static fields, respectively.

The factor $1/\mu_{\eta ,\xi }$ represents spatial correlations,^33^ analogous to 1/ν in temporal correlations. Due to the separability of spatial and temporal variables in the autocorrelation, the space-temporal contrast can be expressed as

$$K^{2}(N,p,x,L,S)=\alpha K_{s}^{2}(N,p)\times K_{t}^{2}(x,L,S)=(\frac{1}{\mu_{0,0}}-\frac{1}{N(N-1)}(4\overset{p}{\underset{\eta =1}{\sum }}(\sqrt{N}-\eta )\sqrt{N}\frac{1}{\mu_{\eta ,0}}+4\overset{p}{\underset{\eta =1}{\sum }}(\sqrt{N}-\eta {)}^{2}\frac{1}{\mu_{\eta ,\eta }}+8\overset{p-1}{\underset{\xi =1}{\sum }}\overset{p}{\underset{\eta =\xi +1}{\sum }}(\sqrt{N}-\eta )(\sqrt{N}-\xi )\frac{1}{\mu_{\eta ,\xi }}))\times K_{t}^{2}(x,L,S)+C_{n}.$$ (15)

The space-time contrast $K^{2}$ consists of three terms. The first term, α, accounts for factors such as polarization and coherence properties of the light source.^21^ The second term corresponds to the spatial correlation $K_{s}^{2}$ [33], whereas the third term corresponds to the temporal correlation $K_{t}^{2}$ term derived from Gaussian or Lorentzian models as described above.

When no spatial correlation is present, the size of the subregion where the pixels may be correlated, which is denoted by p,^33^ equals zero. In this case, the space-time contrast Eq. (15) simplifies to

$$K^{2}(N,0,x,L,S)=K_{s}^{2}(N,0)\times K_{t}^{2}(x,L,S)=\frac{1}{\mu_{0,0}}\times K_{t}^{2}(x,L,S)\;=\frac{1}{\pi^{2}M^{2}}(-1+e^{-\pi M}+\pi \sqrt{M}Erf[\sqrt{\pi M}]{)}^{2}\times K_{t}^{2}(x,L,S),$$ (16)

which is the well-known standard contrast equation incorporating pixel size, as reported previously.^33^^,^^44^

It has been shown that the signal-to-noise ratio improves when using a calibration method that accounts for multiple speckles per pixel^33^^,^^45^^,^^46^ To meet the Nyquist–Shannon criterion, the speckle grain size must be at least twice the pixel size (M≤1/2), and the correction factor $\beta^{1/2}$ depends on the sliding window size (N) and the correlation subregion size (p) where the pixels may be correlated.^33^ Table 1 provides $\beta^{1/2}$ values for sliding windows of 3×3, 5×5, 7×7 and 9×9 pixels with corresponding p values (1, 2, 3, and 4) for Gaussian and Lorentzian correlations, calculated at M=0.5.

**Table 1:** Value of $\beta^{1/2}$ which satisfies the Nyquist-Shannon criterion (M=0.5), for sliding windows of 3×3, 5×5, 7×7, and 9×9  pixels, considering that all the sliding windows may be correlated.

$\beta^{1/2}$ for Nyquist–Shannon criterion	Gaussian correlation	Lorentzian correlation
$N=3^{2}$, p=1	0.73853	0.59253
$N=5^{2}$, p=2	0.77525	0.62463
$N=7^{2}$, p=3	0.78748	0.63846
$N=9^{2}$, p=4	0.7929	0.64521

There are several methods for determining the correlation time $\tau_{c}$, including multi-exposure speckle imaging^21^ and fitting the autocorrelation curve Fig. 8, among others.

## 5 Conclusion

In this study, we developed a mathematical model to accurately calculate temporal speckle contrast, especially for slow-dynamic samples, incorporating correlations between neighboring pixels over time. The model was validated using *E. coli* ATCC 25922 colonies, showing strong agreement with experimental data. It also accommodates fast-dynamic samples and offers analytical solutions for both Gaussian and Lorentzian correlation functions, allowing researchers to choose the most suitable approach for their sample characteristics.

## 6 Appendix: Mathematical Formulation of Temporal Speckle Contrast

Let us consider a set of L frames with exposure time T and a time delay δ between frames (Fig. 1). As mentioned previously in this work, we consider any S consecutive frames to be correlated, i.e., two frames will be considered correlated if there are no more than S frames between them.

The integrated intensity $I_{\psi }$ over the exposure time T of the camera is

$$I_{\psi }=\frac{1}{T}\int_{\psi (T+\delta )}^{(\psi +1)T+\psi \delta }I(t)dt,$$ (17)

then, the mean intensity over the L frames considering L>0 is

$$i=\frac{1}{L}\underset{\psi }{\sum }I_{\psi }.$$ (18)

We note that when L=0, there is only one frame and hence the calculation of temporal contrast is not possible. The corresponding unbiased variance is written as

$$c=\frac{1}{L-1}\overset{L-1}{\underset{\psi =0}{\sum }}(I_{\psi }-i{)}^{2}=\frac{1}{L-1}(-i^{2}L+\overset{L-1}{\underset{\psi =0}{\sum }}I_{\psi }^{2}).$$ (19)

Equation (19) is not defined for L=1. Henceforth, L>1 is considered. Normally, at least 15 frames are used (L=14).

The expected value of the variance is

$$\langle c\rangle =\frac{1}{L-1}(-\langle i^{2}\rangle L+\underset{\psi }{\sum }\langle I_{\psi }^{2}\rangle ),$$ (20)

where ⟨ ⟩ denotes the ensemble average. The parameter $\nu_{\psi }$ depends on the ratio of the exposure time T and correlation time $\tau_{c}$ and the temporal correlation between the frames 0 and ψ.

The correlation factor between two frames separated by ψ frames, denoted by $1/\nu_{\psi }$, is given by the expression

$$\frac{1}{\nu_{\psi }}=\frac{1}{T^{2}}\int_{0}^{T}\int_{\psi (T+\delta )}^{(\psi +1)T+\psi \delta }(g_{2}(t-t^{\prime })-1)dt^{\prime }\;dt=\frac{1}{T^{2}}\int_{0}^{T}\int_{\psi (T+\delta )}^{(\psi +1)T+\psi \delta }g_{1}^{2}(t-t^{\prime })dt^{\prime }\;dt.$$ (21)

$\left\langle i^{2}\right\rangle$ is calculated with Eq. (18), and the summation goes over the L frames is considered. Using the notation of Eq. (23)

$$\langle i^{2}\rangle =\frac{1}{L^{2}}\overset{L-1}{\underset{\psi =0}{\sum }}\overset{L-1}{\underset{\psi^{\prime }=0}{\sum }}\langle I_{0},I_{\psi }\rangle .$$ (22)

In addition, using Eqs. (17) and (21), we write $\left\langle I_{0}I_{\psi }\right\rangle$ as

$$\langle I_{0}I_{\psi }\rangle =\langle I^{2}\rangle (1+\frac{1}{\nu_{\psi }}),$$ (23)

where ⟨I⟩ is the ensemble average of the intensity.

For temporal correlation, there are three types of correlation. Following the notation from our previous work on the first part of our contrast model,^33^ we refer to them as *central* (autocorrelation), *lateral* (in time, correlation between neighbors frames closer than S frames), and the *outsiders* (uncorrelated frames, i.e., correlation between frames that are farther than S frames apart from each other). Then, Eq. (22) may be rewritten as the sum of each type of correlation, i.e.,

$$\langle i^{2}\rangle =\frac{1}{L^{2}}(\underset{\text{central}}{\sum }\langle I_{0},I_{\psi }\rangle +\underset{\text{lateral}}{\sum }\langle I_{0},I_{\psi }\rangle +\underset{\text{outsiders}}{\sum }\langle I_{0},I_{\psi }\rangle ).$$ (24)

We note that, if S=0, there are no lateral type correlations, only the central correlation. Therefore, only the first summation Eq. (23) is not zero. Henceforth, S≥1 is considered to avoid problems with the summation index.

We note that there are L central correlations, one for each pixel of the frames under consideration. The lateral correlation is not so straightforward, but when properly considered, it can be proven that there are 2(L−ψ) equivalent correlations. Defining B(S) as the number of outsider correlations

$$\langle i^{2}\rangle =\frac{1}{L^{2}}(L\langle I_{0}I_{0}\rangle +\overset{S}{\underset{\psi =1}{\sum }}2(L-\psi )\langle I_{0}I_{\psi }\rangle +B(S)\langle I_{0}I_{\psi }\rangle ).$$ (25)

The number of *outsider* correlations can be obtained by subtracting from the total number of correlations, $L^{2}$, the total number of the central and lateral correlations

$$B(S)=L^{2}-(L+\overset{S}{\underset{\psi =1}{\sum }}2(L-\psi ))=L^{2}-L-2SL+S(S+1)\;=(L-S-1)(L-S).$$ (26)

All of these pixels are considered uncorrelated because there are more than S frames between the frames. For those pixels, $\langle I_{0},I_{\psi }\rangle =\langle I_{0}\rangle \langle I_{\psi }\rangle ={\left\langle I\right\rangle }^{2}$. Using Eqs. (23) and (26),

$$\langle i^{2}\rangle =\frac{1}{L^{2}}(L{\left\langle I\right\rangle }^{2}(1+\frac{1}{\nu_{0}})+\overset{S}{\underset{\psi =1}{\sum }}2(L-\psi ){\left\langle I\right\rangle }^{2}(1+\frac{1}{\nu_{\psi }})+B(S){\left\langle I\right\rangle }^{2}).$$ (27)

Factoring ${\left\langle I\right\rangle }^{2}$ and regrouping terms

$$\langle i^{2}\rangle =\frac{{\left\langle I\right\rangle }^{2}}{L^{2}}(L\frac{1}{\nu_{0}}+\overset{S}{\underset{\psi =1}{\sum }}2(L-\psi )\frac{1}{\nu_{\psi }}+(L+\overset{S}{\underset{\psi =1}{\sum }}2(L-\psi ))+B(S)),$$ (28)

by substituting B(S) from Eq. (26) and simplifying the terms, Eq. (28) becomes

$$\langle i^{2}\rangle =\frac{{\left\langle I\right\rangle }^{2}}{L^{2}}(L\frac{1}{\nu_{0}}+\overset{S}{\underset{\psi =1}{\sum }}2(L-\psi )\frac{1}{\nu_{\psi }}+L^{2}).$$ (29)

In a similar way, to compute ⟨c⟩ [Eq. (20)], we note that the term $\left\langle I_{\psi }^{2}\rangle =\langle I_{\psi }I_{\psi }\right\rangle$ is equivalent to $\left\langle I_{\psi }I_{\psi }\rangle =\langle I_{0}I_{0}\right\rangle$. Using Eq. (23), Eq. (20) is rewritten to

$$\langle c\rangle =\frac{1}{L-1}(-\langle i^{2}\rangle L+\underset{\psi }{\sum }{\left\langle I\right\rangle }^{2}(1+\frac{1}{\nu_{0}})).$$ (30)

Simplifying the summation

$$\langle c\rangle =\frac{1}{L-1}(-\langle i^{2}\rangle L+{\left\langle I\right\rangle }^{2}(1+\frac{1}{\nu_{0}})L).$$ (31)

Substituting ${\left\langle i\right\rangle }^{2}$ from Eq. (29) and factoring ${\left\langle I\right\rangle }^{2}$

$$\langle c\rangle =\frac{{\left\langle I\right\rangle }^{2}}{L-1}(-\frac{1}{L^{2}}(L\frac{1}{\nu_{0}}+\overset{S}{\underset{\psi =1}{\sum }}2(L-\psi )\frac{1}{\nu_{\psi }}+L^{2})L+(1+\frac{1}{\nu_{0}})L).$$ (32)

Simplifying Eq. (32)

$$\langle c\rangle ={\left\langle I\right\rangle }^{2}(\frac{1}{\nu_{0}}-\frac{2}{L(L-1)}\overset{S}{\underset{\psi =1}{\sum }}(L-\psi )\frac{1}{\nu_{\psi }}).$$ (33)

Henceforth, S≥1 is considered to avoid problems with the summation index. For the case of S=0, there is only the autocorrelation terms, which means $\langle c\rangle ={\left\langle I\right\rangle }^{2}/\nu_{0}$, and therefore, $K_{t}^{2}(x,L,0)=\frac{1}{\nu_{0}}$. As final step, dividing ⟨c⟩ by ${\left\langle I\right\rangle }^{2}$ to calculate the temporal contrast $K^{2}=\langle c\rangle /{\left\langle I\right\rangle }^{2}$

$$K_{t}^{2}(x,L,S)=\frac{1}{\nu_{0}}-\frac{2}{L(L-1)}\overset{S}{\underset{\psi =1}{\sum }}(L-\psi )\frac{1}{\nu_{\psi }}.$$ (34)

It is important to note that Eq. (34) is an analytic expression for the temporal speckle contrast with an arbitrary number of frames L and an arbitrary number of correlation frames S. No consideration about the intensity distribution $\left(g_{1}^{2}(t-t^{\prime })\right)$ has been made so far.

The temporal contrast [Eq. (34)] is in terms of the factors $1/\nu_{\psi }$. Those factors are given by Eq. (21), which may be simplified using the change of variable $\tau =t-t^{\prime }$, $\overset{¯}{\tau }=(t+t^{\prime })/2$ equivalent to the triangular weight reported in the literature after Bandyopadhyay et al.^30^ Equation (21) is rewritten as

$$\frac{1}{\nu_{\psi }}=\frac{1}{T^{2}}(\int_{(\psi -1)T+\psi \delta }^{\psi T+\psi \delta }(T-T\psi -\delta \psi +\tau )g_{1}^{2}(\tau )d\tau \;+\int_{\psi T+\psi \delta }^{(\psi +1)T+\psi \delta }(T+T\psi +\delta \psi -\tau )g_{1}^{2}(\tau )d\tau ).$$ (35)

We note that no assumptions over $g_{1}^{2}(\tau )$ have been made so far.

### 6.1 Gaussian Correlation

$$g_{1}(\tau )=\exp[-\frac{\pi }{2}\frac{\tau^{2}}{t_{c}^{2}}].$$ (36)

Equation (36) satisfies Mandel’s definition of the correlation time $t_{c}$ (37)

$$t_{c}=\int_{-\infty }^{\infty }|g_{1}(\tau ){|}^{2}d\tau .$$ (37)

The integration on Eq. (35), it is straightforward but computationally expensive

$$\frac{1}{\nu_{\psi }}=\frac{1}{2x}\{(\mathrm{erf}[\sqrt{\pi }(\psi +\psi \Delta )x]-\mathrm{erf}[\sqrt{\pi }(\psi -1+\psi \Delta )x])(1-\psi -\psi \Delta )\;+(\mathrm{erf}[\sqrt{\pi }(\psi +1+\psi \Delta )x]-\mathrm{erf}[\sqrt{\pi }(\psi +\psi \Delta )x])(1+\psi +\psi \Delta )\}\;+\frac{1}{2\pi x^{2}}(\exp[-\pi (\psi -1+\psi \Delta {)}^{2}x^{2}]-2\;\exp[-\pi (\psi +\psi \Delta {)}^{2}x^{2}]\;+\exp[-\pi (\psi +1+\psi \Delta {)}^{2}x^{2}]),$$ (38)

where Δ=δ/T and $x=T/\tau_{c}$.

### 6.2 Lorentzian Correlation

$$g_{1}(\tau )=\exp[-\frac{\left|\tau \right|}{t_{c}}].$$ (39)

Equation (39) also satisfies Mandel’s definition of the correlation time Eq. (37).

Due to the absolute value, it is necessary to be careful with the limits on the integrals of Eq. (35)

$$l_{1}=(\psi -1)T+\psi \delta ,\;l_{2}=\psi T+\psi \delta ,\;l_{3}=(\psi +1)T+\psi \delta .$$ (40)

T and δ are times, and therefore, they are always positive. For ψ∈N, $l_{2},l_{3}>0$ for all T,δ,ψ. Only $l_{1}$ could be negative when ψ<1⇒ψ=0. For this reason, Eq. (35) is solved and the solution is presented in two cases, for ψ=0 and ψ>0.

For ψ=0

$$\frac{1}{\nu_{0}}=\frac{1}{2x^{2}}(2x+\exp[-2x]-1).$$ (41)

For ψ>0

$$\frac{1}{\nu_{\psi }}=\frac{1}{4x^{2}}(-2\;\exp[-2(\psi +\psi \Delta )x]+\exp[-2((\psi -1)+\psi \Delta )x]\;+\exp[-2((\psi +1)+\psi \Delta )x]),$$ (42)

where Δ=δ/T and $x=T/\tau_{c}$.

## Data Availability

Implementation of Eqs. (4), (8), (11), and (12) as functions in Mathematica and MATLAB is available at the GitHub repository: *TemporalSpeckle*.^47^ The necessaries functions for the spatial contrast is also available in GitHub; *SpatialSpeckle*.^48^
